# Omega-3 Fatty Acids: Possible Neuroprotective Mechanisms in the Model of Global Ischemia in Rats

**DOI:** 10.1155/2016/6462120

**Published:** 2016-05-24

**Authors:** Maria Elizabeth Pereira Nobre, Alyne Oliveira Correia, Francisco Nilson Maciel Mendonça, Luiz Ricardo Araújo Uchoa, Jessica Tamara Nunes Vasconcelos, Carlos Ney Alencar de Araújo, Gerly Anne de Castro Brito, Rafaelly Maria Pinheiro Siqueira, Gilberto dos Santos Cerqueira, Kelly Rose Tavares Neves, Ricardo Mário Arida, Glauce Socorro de Barros Viana

**Affiliations:** ^1^Faculty of Medicine, Estácio of Juazeiro do Norte (FMJ), Rua Tenente Raimundo Rocha 515, 63040-360 Juazeiro do Norte, CE, Brazil; ^2^Federal University of Ceará (UFC), Rua Coronel Nunes de Melo 1127, 60430-270 Fortaleza, CE, Brazil; ^3^Federal University of São Paulo (UNIFESP), Rua Pedro de Toledo 669, 04039-032 São Paulo, SP, Brazil

## Abstract

*Background*. Omega-3 (ω3) administration was shown to protect against hypoxic-ischemic injury. The objectives were to study the neuroprotective effects of ω3, in a model of global ischemia.* Methods*. Male Wistar rats were subjected to carotid occlusion (30 min), followed by reperfusion. The groups were SO, untreated ischemic and ischemic treated rats with ω3 (5 and 10 mg/kg, 7 days). The SO and untreated ischemic animals were orally treated with 1% cremophor and, 1 h after the last administration, they were behaviorally tested and euthanized for neurochemical (DA, DOPAC, and NE determinations), histological (Fluoro jade staining), and immunohistochemical (TNF-alpha, COX-2 and iNOS) evaluations. The data were analyzed by ANOVA and Newman-Keuls as the* post hoc* test.* Results*. Ischemia increased the locomotor activity and rearing behavior that were partly reversed by ω3. Ischemia decreased striatal DA and DOPAC contents and increased NE contents, effects reversed by ω3. This drug protected hippocampal neuron degeneration, as observed by Fluoro-Jade staining, and the increased immunostainings for TNF-alpha, COX-2, and iNOS were partly or totally blocked by ω3.* Conclusion*. This study showed a neuroprotective effect of ω3, in great part due to its anti-inflammatory properties, stimulating translational studies focusing on its use in clinic for stroke managing.

## 1. Background

Ischemic stroke is a pathologic condition and a major cause of death and disability worldwide. Because of its huge socioeconomic burden and considering the global life expectancy increases, one can assume that stroke is already the most challenging disease [1]. Although animal stroke models have shed light on the pathophysiology of ischemic stroke, the translation of these results from bench to bedside has been somewhat disappointing [2, 3].

The most common cause of stroke is the sudden occlusion of a blood vessel by a thrombus or embolism, resulting in an almost immediate loss of oxygen and glucose to the cerebral tissue. Brain is almost exclusively dependent on the continuous steady flow of glucose and oxygen to undergo oxidative phosphorylation for energy production, since it has no energetic stores. Within minutes of vascular occlusion, a complex sequence of pathophysiological events called ischemic cascade occurs. The first consequence is the depletion of substrates, particularly oxygen and glucose, that causes the accumulation of lactate via anaerobic glycolysis. Acidosis may enhance free-radical formation, interfering with intracellular protein synthesis and worsening ischemic brain injury [4, 5]. Energy failure leads to perturbation of the Na^+^/K^+^-ATPases and Ca^2+^/H-ATPases pumps and, in addition, the Na^+^-Ca^2+^ transporter is reversed [6]. Subsequently, an alteration of ion homeostasis causes cytotoxic edema, leading to events triggered by intracellular Ca^2+^ excess [1].

*ω*3 fatty acids are a group of essential fatty acids that serve as energy substrates and integral membrane components and, therefore, play crucial roles in the maintenance of normal neurological functions. Recent studies show that *ω*3 fatty acids display neuroprotective properties and may exert beneficial effects on cerebral ischemia and other brain disorders [7]. It is well recognized that cerebral ischemia induces excessive release of excitatory amino acids, as glutamate and aspartate, which provoke enzymatic processes leading to irreversible neuronal injury [8]. On the other hand, several years ago, the behavioral effects of *ω*3 fatty acids deficiency were proposed to be mediated through monoaminergic neurotransmission, including the dopaminergic system, what was evidenced years later [9, 10].

Thus, the objectives of the present work were to study the possible neuroprotective effects of *ω*3 fatty acids in a model of global brain ischemia in rats, focusing on behavior and striatal DA, DOPAC, and NE levels. Besides, considering that inflammation plays an important role in the pathogenesis of ischemic stroke [11, 12] and the anti-inflammatory properties of *ω*3 [13–16] also observed by us [17], we decided to analyze the action of *ω*3 fatty acids on proinflammatory cytokines and enzymes, in hippocampi from ischemic animals, through TTC and Fluoro-Jade staining and with immunohistochemical assays.

## 2. Material and Methods

### 2.1. Drugs and Reagents

Proepa (Aché Laboratórios Farmacêuticos SA) was the source of *ω*3 fatty acids, while sodium thiopental was from Laboratório Cristália, Brazil. Cremophor EL, 2,3,5-triphenyltetrazolium chloride (TTC), standard monoamines, and standard amino acids were from Sigma-Aldrich, USA. All other reagents were of analytical grade.

### 2.2. Animals

Male Wistar rats (200–250 g) were obtained from the Animal House of the Faculty of Medicine Estácio of Juazeiro do Norte, Brazil. The animals were housed at 24 ± 2°C, under a 12 h light/12 h dark cycle and had free access to a standard pellet diet (Purina chow) and tap water. They were deprived of food for 8 h, before the experiments, except for drinking water. The animals were treated in accordance with the current law and the NIH Guide for the Care and Use of Laboratory Animals. The project was previously approved by the Animal's Ethics Committee of the Faculty of Medicine of the Federal University of Ceará at the city of Barbalha, Brazil.

### 2.3. Experimental Protocol

The animals were anesthetized with sodium thiopental (50 mg/kg, i.p.) and submitted to the model of transitory global ischemia, by the bilateral occlusion of both carotids, for 30 min. After reperfusion, the incision was sutured and the animals placed in individual plastic cages for recovery, with water and food* ad libitum*. The sham-operated groups (SO, controls) were submitted to the same procedure, except for the clamping of the carotids. After surgery, the animals were orally treated (by gavage) with *ω*3 fatty acids, at the doses of 5 and 10 mg/kg, daily for 7 days. The SO and ischemic groups received 1% Cremophor (1 mL/kg). The animals were distributed into 4 groups (except for COX-2 immunostaining, where the *ω*3 dose of 2.5 mg/kg was also used) as follows: SO, untreated ischemic, and ischemic groups after treatments with *ω*3 (5 and 10 mg/kg, p.o.). At the 7th day of treatment and 1 h after the last drug administration, the animals were submitted to behavioral tests (open field and water maze tests) and sacrificed for* striata* dissection and hippocampal slicing. The* striata* were used for homogenate preparations and DA, DOPAC, and NE determinations. Hippocampal slices were processed for immunohistochemistry assays.

### 2.4. Behavioral Testing

#### 2.4.1. Open Field Test

This test is used to measure locomotor activity in rodents and can also serve to determine motor deficits and anxiety. It was originally described for the study of emotionality in rats and now is one of the most popular models in animal psychology [18]. Locomotor activity is measured by determining the amount of distance traveled and stereotyped behaviors, as rearing and grooming. The test is sensitive to motor dysfunction, as well as hippocampal and basal ganglia damage, and we used an arena (50 × 50 × 20 cm) divided into four equal quadrants. Each animal was evaluated for 5 min, at the 7th day after ischemia, and we determined the number of quadrants crossed by the animal with all 4 paws.

#### 2.4.2. Morris Water Maze Test

This is a test of spatial learning for rodents that relies on distal cues to navigate from start locations around the perimeter of an open swimming arena for locating a submerged escape platform. The original procedure was described as Morris water maze [19, 20]. Spatial learning is assessed across repeated trials and reference memory. The test has proven to be a robust and reliable one and is strongly correlated with hippocampal synaptic plasticity and NMDA receptor function. For that, we used a circular black pool (1.7 m of diameter and 1 m height), filled with water (0.59 m deep), at 25°C temperature. The pool is divided into four quadrants and has a 10 cm diameter platform immersed 0.5 cm below the water surface. The room is provided with four cues located on North, South, East, and West of the walls. The animals were subjected to two trials, for two consecutive days (pretraining), and, 48 h after the last trial, to the test in the water maze. The animals had a maximum time of 54 s (cut-off time) for finding the platform, where they can stay for 15 s but would have a second chance, in case of failure.

#### 2.4.3. Catecholamine Determinations by HPLC

For measurements of dopamine (DA) and its metabolite (3,4-dihydroxyphenylacetic acid, DOPAC) as well as noradrenaline (NE),* striata* from all groups were used to prepare 10% homogenates. Homogenates were sonicated in 0.1 M HClO_4_, for 30 s, and centrifuged at 4°C for 15 min at 15,000 rpm, and the supernatants were filtered (0.2 *μ*m, Millipore). Twenty-microliter samples were then injected into a high-performance liquid chromatograph (HPLC) column. The mobile phase was 0.163 M citric acid, pH 3.0, containing 0.02 mM EDTA with 0.69 mM sodium octanesulfonic acid (SOS), as an ion pairing reagent, 4%  v/v acetonitrile, and 1.7%  v/v tetrahydrofuran. The monoamines were electrochemically detected, using an amperometric detector (Shimadzu, Japan), by oxidation on a glassy carbon electrode at 0.85 V relative to the Ag-AgCl reference electrode. Their concentrations were determined by comparison with standards injected into the HPLC column at the day of experiment and the values expressed as ng/g tissue.

#### 2.4.4. TTC Staining

TTC staining is considered a reliable method for detection of cerebral infarction in rats after ischemia [21]. The animals were decapitated and their brains removed for TTC (2,3,5-triphenyltetrazolium chloride) staining. Then, 2 mm coronal sections were obtained with the rodent brain matrix apparatus (Harvard, USA). The slices were incubated with TTC for 30 min at 37°C in the dark and fixed by immersion in 4% buffered (7.4) formaldehyde solution for 24 h. Afterwards, the sections were photographed and ischemic areas were quantified by the Image J software (NIH, USA). In those cells populations where the electron mitochondrial transport is maintained, the TTC shows a red color that is greatly decreased in brain ischemic areas.

#### 2.4.5. Fluoro-Jade Staining

Fluoro-Jade is an anionic fluorescein derivative, used for the histological staining of neurons which become fluorescent after degeneration. After paraffin removal (by immersion in xylol), hippocampus sections (5 *μ*m) were mounted on slides surrounded by gelatin and rehydrated by immersion in ethanol for 3 min, followed by immersions in 70 and 50% ethanol solutions and distilled water. The slices were placed into a 0.06% potassium permanganate solution, for 15 min, washed in distilled water, and transferred to a Fluoro-Jade solution where they stayed for 30 min (with gentle stirring). After staining, the slices were washed in distilled water (3 times, 2 min each time). The excess of water was discarded and the dry slices mounted in Fluoromount® media and examined with a fluorescence microscope. The data were quantified by the Image J software (NIH, USA).

#### 2.4.6. Immunohistochemistry Assays for TNF-Alpha, COX-2, and iNOS

Brain hippocampal sections were fixed in 10% buffered formol, for 24 h, followed by a 70% alcohol solution and embedded into paraffin wax for slices processing on appropriate glass slides. These were placed into the oven at 58°C, for 10 min, followed by deparaffinization in xylol, rehydration in alcohol at decreasing concentrations, and washing in distilled water and PBS (0.1 M sodium phosphate buffer, pH 7.2), for 10 min. The endogenous peroxidase was blocked with a 3% hydrogen peroxide solution, followed by incubation with the appropriate primary anti-antibody for TNF-alpha, iNOS, and COX-2, and diluted according to the manufacturers' instructions (Santa Cruz or Millipore, USA), for 2 h, at room temperature in a moist chamber. The glass slides were then washed with PBS (3 times, 5 min each) and incubated with the biotinylated secondary antibody, for 1 h, at room temperature in a moist chamber. Then, they were washed again in PBS and incubated with streptavidin-peroxidase, for 30 min, at room temperature (in a moist chamber) and, after a final wash in PBS, incubated in 0.1% DAB solution (in 3% hydrogen peroxide). Finally, the glass slides were washed in distilled water and counterstained with Mayer's hematoxylin, washed in tap water, dehydrated in alcohol (at increasing concentrations), diaphonized in xylol, and mounted on Entelan® for optic microscopy examination. The data were quantified by the Image J software (NIH, USA).

### 2.5. Statistical Analyses

The data are presented as means ± SEM and analyzed by one-way ANOVA, followed by Newman-Keuls test as the* post hoc* test. Whenever needed the data were analyzed by two-tailed unpaired Student's *t*-test. The differences were considered statistically significant at *p* < 0.05.

## 3. Results

### 3.1. Behavioral Tests

#### 3.1.1. Open Field Test

**Figure 1:**
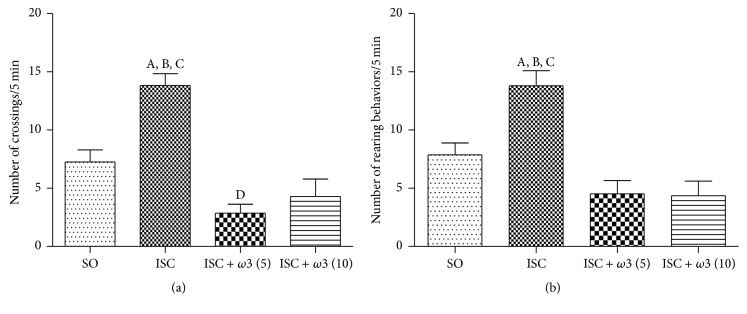
Evaluations of the number of crossing (a) and rearing (b) behaviors by the open field test. The columns represent means ± SEM (number of crossings/5 min) from SO and rats subjected to cerebral ischemia untreated or after oral treatments with *ω*3, at the doses of 5 and 10 mg/kg (number of animals per group: SO = 27; ISC = 35; ISC + *ω*3 (5) = 14; ISC + *ω*3 (10) = 17). (a) (A) versus SO, q = 6.591^*∗∗∗*^; (B) versus ISC + *ω*3 (5), q = 8.916^*∗∗∗*^; (C) versus ISC + *ω*3 (10), q = 8.288^*∗∗∗*^; (D) versus SO, q = 3.435^*∗*^. (b) (A) versus SO, q = 5.533^*∗∗∗*^; (B) versus ISC + *ω*3 (5), q = 6.808^*∗∗∗*^; (C) versus ISC + *ω*3 (10), q = 7.246^*∗∗∗*^ (one-way ANOVA and Newman-Keuls test as the* post hoc* test).

We showed a 1.9-fold increase in locomotor activity in the untreated ischemic group, as related to the sham-operated one (SO, controls). The treatment of ischemic groups with *ω*3 (5 and 10 mg/kg, p.o.) significantly reversed the locomotor activity to values close to normality (Figure 1(a)). A similar profile was seen with the rearing behavior (Figure 1(b)). These effects were reversed after *ω*3 treatments and, in both cases, the values were even lower than those of the sham-operated group.

#### 3.1.2. Morris Water Maze Test

**Figure 2:**
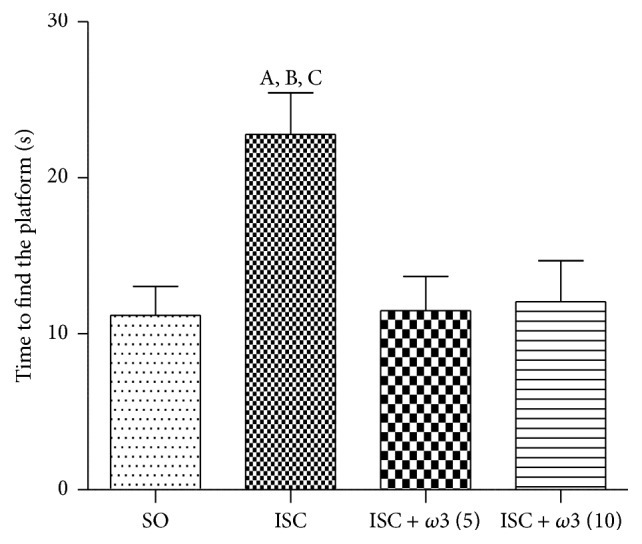
Evaluation of the spatial memory by the water maze test. The columns represent means ± SEM of the time (s) to find the platform. The studied groups were SO and ischemic animals untreated or after oral treatments with *ω*3, at the doses of 5 and 10 mg/kg (number of animals per group: SO = 28; ISC = 27; ISC + *ω*3 (5) = 14; ISC + *ω*3 (10) = 14). (A) versus SO, q = 5.159^*∗∗*^; (B) versus *ω*3 (5), q = 4.016^*∗*^; (C) versus *ω*3 (10), q = 3.897^*∗∗*^ (one-way ANOVA and Newman-Keuls test as the* post hoc* test).

We showed a significant 2-fold increase in the time to find the platform after ischemia, as related to controls (SO), indicating an impairment of spatial learning and hipocampal dysfunction. On the other hand, the repeated treatment of ischemic groups with *ω*3 (5 and 10 mg/kg), for 7 days, completely reversed the effects, showing an improvement on spatial memory due to *ω*3 treatments (Figure 2).

### 3.2. Neurochemical Determinations

#### 3.2.1. Dopamine (DA), DOPAC, and NE Determinations in Rat Brain Striatum

**Figure 3:**
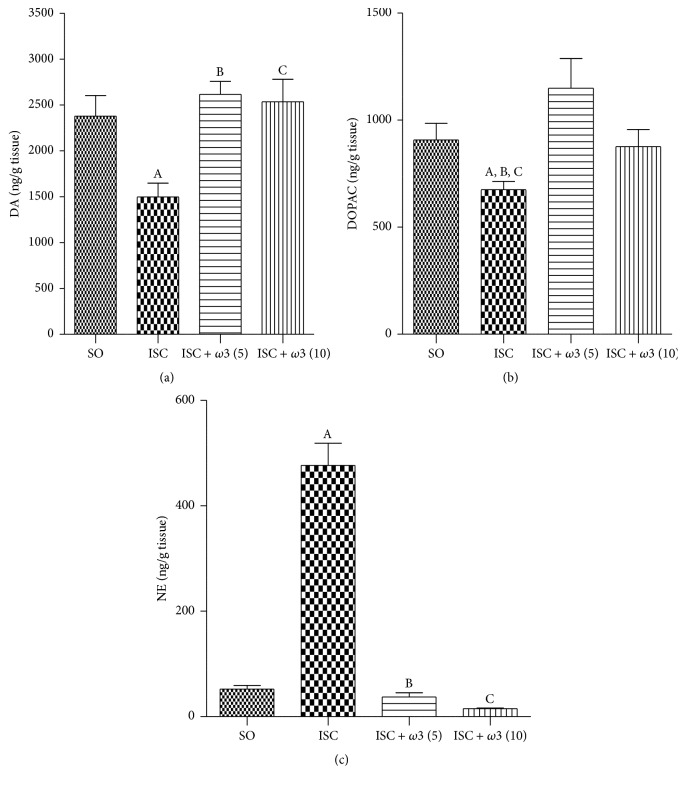
Evaluation of *ω*3 treatments at the doses of 5 and 10 mg/kg on the striatal contents of DA (a), DOPAC (b), and NE (c), in rats subjected to brain ischemia and reperfusion for 7 days. The numbers of animals per group were the following: for DA, SO = 14, ISC = 9, ISC + *ω*3 (5) = 19, and ISC + *ω*3 (10) = 15; for DOPAC, SO = 14, ISC = 11, ISC + *ω*3 (5) = 15, and ISC + *ω*3 (10) = 14; for NE, SO = 8, ISC = 10, ISC + *ω*3 (5) = 8, and ISC + *ω*3 (10) = 6. The columns represent means ± SEM and the groups are sham-operated (SO); ischemic (ISC) untreated; and ischemic after *ω*3 treatments for 7 days. DA: (A) versus SO, q = 3.830^*∗∗*^; (B) versus *ω*3 (5), q = 5.145^*∗∗*^; (C) versus *ω*3 (10), q = 4.570^*∗∗*^. DOPAC: (A) versus SO, t = 2.484, df = 23; (B) versus *ω*3 (5), q = 4.707^*∗∗*^; (C) versus *ω*3 (10), t = 2.085, df = 23. NE: (A) versus SO, q = 16.52^*∗∗∗*^; (B) versus *ω*3 (2.5), q = 18.79^*∗∗∗*^; (C) versus *ω*3 (5), q = 17.09^*∗∗∗*^ (one-way ANOVA and Newman-Keuls test as the* post hoc* test).

Our results showed that brain ischemia decreases in 37% DA levels, as related to controls (SO) (Figure 3(a)). On the other hand, DA values returned to normality (and similarity to those of the SO group), in the ischemic group treated for 7 days with *ω*3 (5 and 10 mg/kg, p.o.). Similar results were observed with DOPAC where a 26% decrease was seen in the ischemic untreated group, as related to controls (SO). In both cases, the values in the ischemic group after *ω*3 treatments (Figure 3(b)) returned to those shown by the SO group. Surprisingly, a 9-fold increase in NE contents, as related to the SO group, was observed in the untreated ischemic group. NE levels returned to values even lower than those of the SO group, in ischemic animals after *ω*3 treatments with the doses of 5 and 10 mg/kg (Figure 3(c)).

### 3.3. Histological and Immunohistochemistry Assays

#### 3.3.1. TTC Staining in the Hippocampus

**Figure 4:**
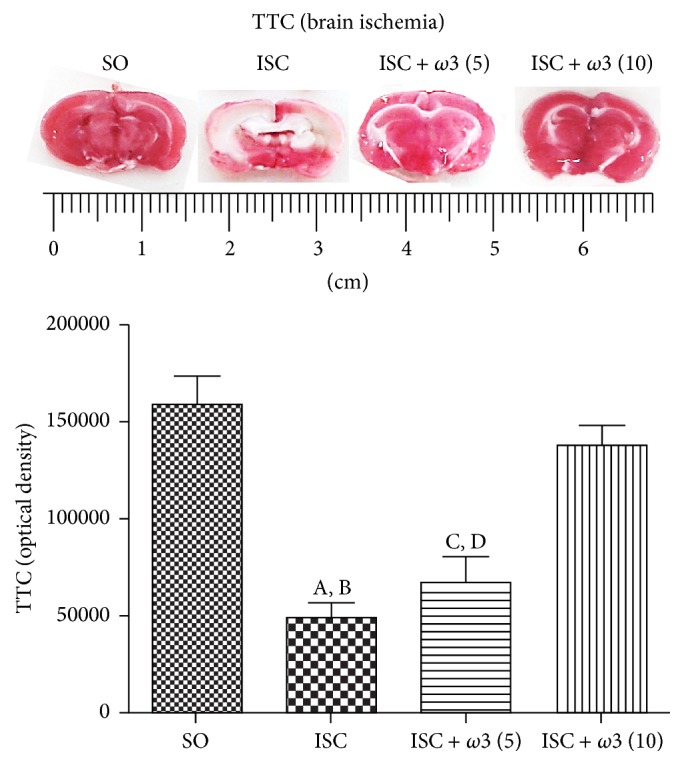
Effects of *ω*3 treatments in ischemic groups, visualized by the 2,3,5-triphenyltetrazolium chloride (TTC) staining as related to the SO group. (A) versus SO, q = 9.546^*∗∗∗*^; (B) versus ISC + *ω*3 (10), q = 8.194^*∗∗∗*^; (C) versus SO, q = 7.894^*∗∗∗*^; (D) versus ISC + *ω*3 (10), q = 6.452^*∗∗∗*^ (one-way ANOVA and Newman-Keuls test as the* post hoc* test).

The ischemic group (ISC) showed a 69% decrease in TTC staining as related to the SO group. This decrease was of only 13% in the ischemic group after *ω*3 treatment with the dose of 10 mg/kg (ISC + *ω*3 (10)). On the other hand, no significant differences were observed in the ISC group (ISC) without and after treatment with *ω*3 at the dose of 5 mg/kg (ISC + *ω*3 (5)) (Figure 4).

#### 3.3.2. Fluoro-Jade Staining in the Hippocampus

**Figure 5:**
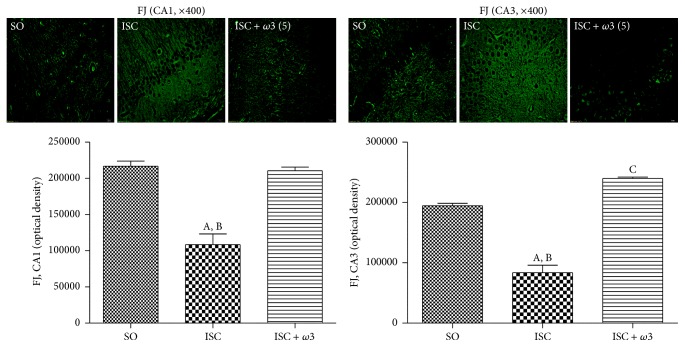
*ω*3 treatments of ischemic animals (3 animals per group) drastically decreased the neuronal degeneration (visualized by an intense fluorescence to Fluoro-Jade) in hippocampus and cortex. The effects in CA1 and CA3 and DG are represented by photomicrographs and quantitative measurements performed with the Image J software, from 3 to 6 fields. The groups are sham-operated (SO); ischemic (ISC) untreated, and ischemic after *ω*3 treatments (5 mg/kg, for 7 days). CA1: (A) versus SO, q = 10.98^*∗∗∗*^; (B) versus ISC + *ω*3, q = 10.34^*∗∗∗*^. CA3: (A) versus SO, q = 14.57^*∗∗∗*^; (B) versus ISC + *ω*3, q = 20.55^*∗∗∗*^; (C) versus SO, q = 5.979^*∗∗*^ (one-way ANOVA and Newman-Keuls test as the* post hoc* test).

A greater number of fluorescent cells were observed in the CA1 hippocampal subfield of the untreated ischemic group, indicating neuronal degeneration. This change is quantified by the Image J software as a decrease (50%) in optical density, as related to the SO group whose neurons appear darker. A similar picture was observed in the CA3 area (51% decrease) and in the dentate gyrus (56% decrease). In all cases, these alterations were completely reversed after *ω*3 treatments (Figure 5).

#### 3.3.3. Immunohistochemistry for TNF-Alpha, COX-2, and iNOS in Hippocampus and Temporal Cortex

**Figure 6:**
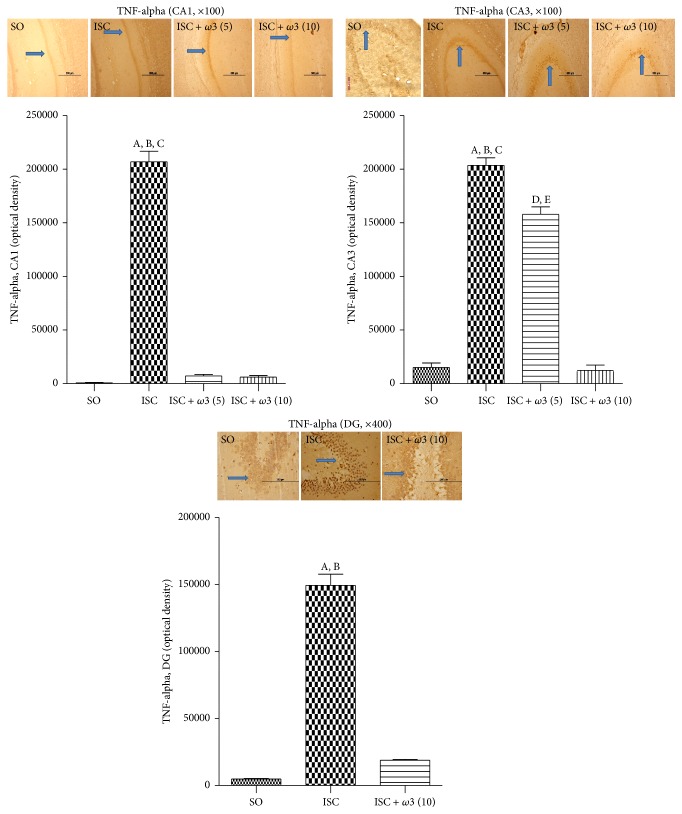
*ω*3 treatments of ischemic animals (3 animals per group) reduced the immunostaining for TNF-alpha, mainly in CA1 and CA3 hippocampal subfields and in the dentate gyrus (DG). Representative photomicrographs and quantitative measurements performed with the Image J software from 3 to 5 fields. The groups are SO, untreated ischemic (ISC) and ischemic after *ω*3 treatments (5 and 10 mg/kg, 7 days). CA1: (A) versus SO, q = 41.76^*∗∗∗*^; (B) versus ISC + *ω*3 (5), q = 40.44^*∗∗∗*^; (C) versus ISC + *ω*3 (10), q = 40.63^*∗∗∗*^. CA3: (A) versus SO, q = 32.86^*∗∗∗*^; (B) versus ISC + *ω*3 (5), q = 7.494^*∗∗∗*^; (C) versus ISC + *ω*3 (10), q = 33.31^*∗∗∗*^; (D) versus SO, q = 7.494^*∗∗∗*^; (E) versus ISC + *ω*3 (10), q = 23.91^*∗∗∗*^. DG: (A) versus SO, q = 30.26^*∗∗∗*^; (B) versus ISC + *ω*3 (10), q = 27.34^*∗∗∗*^ (one-way ANOVA and Newman-Keuls test as the* post hoc* test).

The immunohistochemistry data for TNF-alpha showed a higher number of immunopositive cells in CA1, CA3, and dentate gyrus areas in the ischemic group (ISC) as related to the SO group. The effects were much more intense in the CA1 (313-fold increase), followed by DG (30-fold increase) and CA3 (14-fold increase) areas. In all cases, these changes were significantly decreased after *ω*3 treatments (Figure 6).

**Figure 7:**
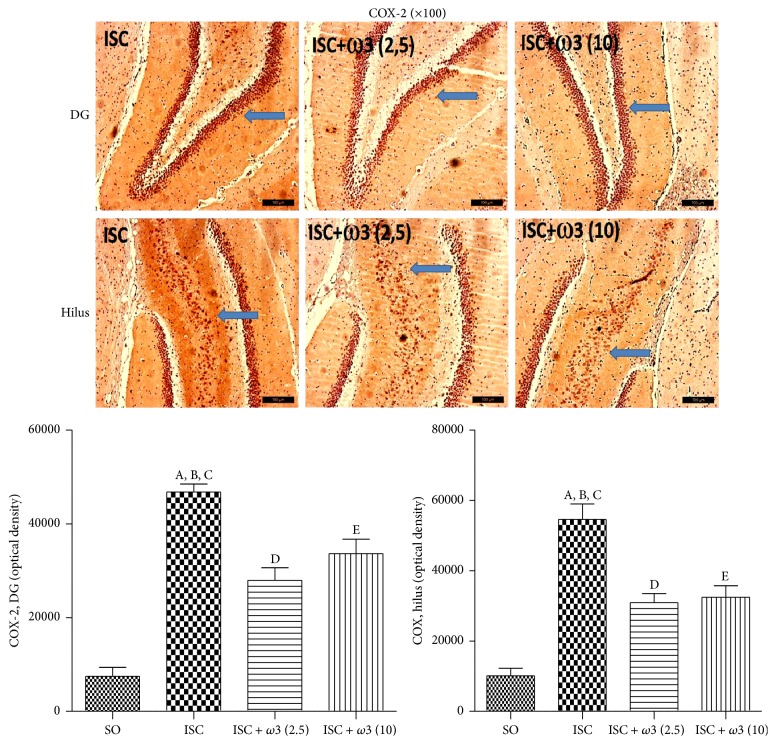
*ω*3 treatments of ischemic animals (3 animals per group) reduced the immunostaining for COX-2, mainly in the dentate gyrus (DG) and hilus. Representative photomicrographs and quantitative measurements performed with the Image J software, from 3 to 5 fields. The groups are untreated ischemic (ISC) and ischemic after treatments with *ω*3 (2.5 and 10 mg/kg, 7 days). DG: (A) versus SO, q = 14.88^*∗∗∗*^; (B) versus ISC + *ω*3 (2.5), q = 7.135^*∗∗∗*^; (C) versus ISC + *ω*3 (10), q = 4.979^*∗∗*^. Hilus: (A) versus SO, q = 13.95^*∗∗∗*^; (B) versus ISC + *ω*3 (2.5), q = 7.418^*∗∗∗*^; (C) versus ISC + *ω*3 (10), q = 6.943^*∗∗∗*^; (D) versus SO, q = 6.527^*∗∗∗*^; (E) versus SO, q = 7.003^*∗∗∗*^ (one-way ANOVA and Newman-Keuls test as the* post hoc* test).

**Figure 8:**
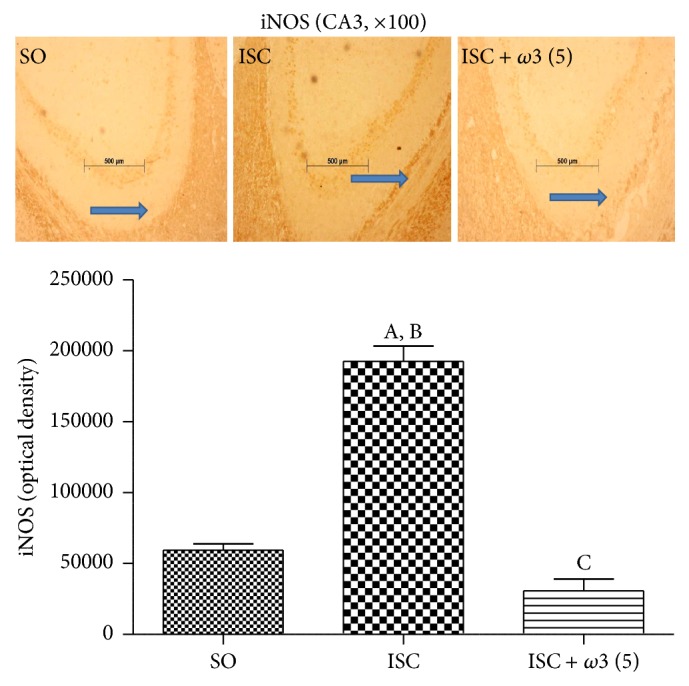
*ω*3 treatments (3 animals per group) reduced the immunostaining for iNOS mainly in the CA3 hippocampal subfield. Representative photomicrographs and measurements performed with the Image J software, from 3 to 5 fields. The groups are sham-operated (SO), untreated ischemic (ISC), and ischemic after *ω*3 treatment (5 mg/kg, 7 days). (A) versus SO, q = 16.03^*∗∗∗*^; (B) versus ISC + *ω*3 (5), q = 19.49^*∗∗∗*^; (C) versus SO, q = 3.456^*∗*^ (one-way ANOVA and Newman-Keuls test as the* post hoc* test).

Figure 7 shows representative photomicrographs for COX-2 immunohistochemistry assays in the dentate gyrus (DG) and hilus. While increases of 6- and 5-fold were demonstrated in the DG and hilus ischemic groups, respectively, these effects were significantly reduced to similar values in both areas after *ω*3 treatments. Increases around 3.9-fold in the iNOS immunoreactivity were detected in the CA3 area from ischemic groups as related to the SO group. The effects were reduced to values even lower than those from the SO group after treatments with *ω*3 at the dose of 5 mg/kg (Figure 8).

## 4. Discussion

Global cerebral ischemia in rodents is characterized morphologically by a selective neuronal damage, particularly in the hippocampus, but also in the striatum and cortex [22]. The resultant damage to vulnerable cells, notably in the CA1 and hilar hippocampal fields, is frequently associated with memory deficits [23]. The most common model for global cerebral ischemia in rodents uses brain ischemia associated with hypotension [24], which results in higher brain damage mainly in the hippocampus. However, in the present work we used the common carotids occlusion for 30 min without hypotension, which has been shown to cause alterations in hippocampal CA1 neurons [25].

Furthermore, animal studies particularly in rodents indicate that long chain *ω*3 fatty acids play a role in behavioral and cognitive functions. Chronic *ω*3 fatty acids deficiency has been shown to increase anxiety in rodents, particularly under stressful conditions [26–28]. It has been demonstrated that a decreased content of docosahexaenoic acid (DHA), a long chain polyunsaturated fatty acid of the *ω*3 series, is accompanied by anxiety and learning and memory impairments that have been associated with changes in neurotransmission processes [10].

Most of the literature data deal with animals chronically submitted to *ω*3 supplemented diet [29–33] or *ω*3 high dose, as seen in preclinical [34, 35] and clinical studies as well [36]. As far as we know, ours is the first study dealing with *ω*3 administration for a short period and at a lower dose range. We focused on behavioral changes, determinations of striatal monoamines, and immunohistochemistry assays in the hippocampus of animals submitted to global brain ischemia untreated or treated with *ω*3 fatty acids.

The majority of studies performed with *ω*3 describe its dietary effects after 3 to 5 weeks [37–39] and very few [40] use a short-term treatment (1 week). While this last study showed an effect of *ω*3 on lipid metabolism after short-term treatment, others [41] observed no effect after the *ω*3 treatment for 2 weeks on the scopolamine-induced amnesia. In the present study we demonstrated that *ω*3 at lower doses can be effective after daily administration by gavage to rats and well absorbed after its emulsification as already shown [42] and in an empty stomach. This short-term effect of lower doses of *ω*3 was clearly observed by us after the TTC staining, a reliable method for the detection of brain ischemic areas in the rat [21].

In the open field test, we showed that after global brain ischemia the animals presented not only an increased locomotor activity, but also an increased number of rearing behaviors, as related to controls (SO). On the other hand, the treatment of ischemic groups with *ω*3 significantly decreased all these behavioral parameters to values close to those of controls. Brain ischemia is known to increase locomotor activity and anxiety [43–47] and the degree of hippocampal damage has been positively correlated with the increase in motor activity.

Our data showed that brain ischemia significantly impaired spatial memory, and this effect was completely reversed after *ω*3 treatments. Evidences indicate that transient global ischemia may lead to severe impairments in learning and memory [48–50]. The pyramidal CA1 neurons of the hippocampus are critically involved in spatial learning and memory and are also especially vulnerable to cerebral ischemia. The transient global cerebral ischemia model results in neurodegeneration, known to induce both hippocampal neuronal loss and learning deficits in rats [48, 50, 51]. Reduction of memory deficits by PUFA, after global ischemia, has been already observed by others [30, 34, 52]. However, in most of these data, the animals were chronically subjected to a rich-in-*ω*3 fatty acids diet, while in the present study the animals were orally given lower doses of *ω*3, for a short period of time.

We also showed a 37% reduction in DA levels in the striata of ischemic rats, as related to the SO group. On the other hand, these effects were completely reverted after *ω*3 treatments and a similar picture was noticed in DOPAC levels. Ischemic conditions can induce the release of brain neurotransmitters. Earlier studies showed that the DA metabolism is significantly altered during and after ischemia in the rabbit retina [53]. On the other hand, biochemical consequences of ischemia in the hippocampus, cortex, and striatum have been object of much attention, because these brain structures are particularly vulnerable to ischemia [54]. The extracellular levels of DA have been shown to increase drastically, immediately after ischemia. This increase is followed by a great decrease, probably reflecting that the release of DA is not compensated by an increased synthesis of this neurotransmitter [55–60].

Evidences indicate that DA release exerts an important role in the striatal damage observed after ischemia. As a matter of fact, the depletion of DA by alpha-methyltyrosine or 6-OHDA before the ischemic process reduces the degree of neuronal damage [61–63], using an experimental model similar to ours, showing by microdialysis that the extracellular concentrations of DA increased abruptly, around 3 min after the ischemic insult, reaching a maximum value between 20 and 40 min after the insult and decreasing subsequently. These authors concluded that, during ischemia, the great increase in DA concentrations occurs probably as a result of energy failure in cellular membranes. Then, the DA release could be the cause of the neuronal damage, associated with brain ischemia.

In our study, it is possible that, because DA determinations in the striatum were carried out 7 days after ischemia, this brain tissue was already partly recovered. Despite that, our results showed a partial recovery of the alterations in DA levels in the ischemic groups after *ω*3 treatments. Others [7] showed that *ω*3 fatty acids present neuroprotective properties, exerting benefits on cognitive functions, neurodegenerative diseases, and brain ischemia.

Evidences [64] have indicated the effects of a diet which was deficient in alpha-linolenic acid, precursor of long chains unsaturated fatty acids as *ω*3, on the dopaminergic neurotransmission in the core of the rat* nucleus accumbens*. These* in vivo* microdialysis experiments showed increased basal levels of dopamine and their metabolites (DOPAC and HVA), in rats subjected to a fatty-acid-deficient diet, as compared to controls, indicating alterations in the dopaminergic neurotransmission in the* nucleus accumbens*.

For the assessment of neuronal degeneration, we used the staining with Fluoro-Jade which is a fluorescein derivative that specifically binds to degenerating neurons. Fluoro-Jade stains cell bodies, dendrites, axons, and their terminals but does not stain healthy neurons [65]. We showed a high number of degenerating neurons in the CA1, CA3,* dentate gyrus*, and temporal cortex from untreated ischemic rats, and this profile was completely reversed after *ω*3 treatments, towards that of SO (controls), indicating the drug neuroprotective effects. Interestingly, Fluoro-Jade staining demonstrating neuronal damage was shown in some brain areas of animals submitted to PUFAs dietary restriction [66], corroborating with our findings.

While PUFAs are known to present brain immunomodulatory properties, n-3 PUFAs are able to reduce inflammation and n-6 PUFAs are more proinflammatory [67]. These authors showed that the central n-3 PUFA increase modulates the brain innate immune system, leading to the protection against LPS-induced proinflammatory cytokine production and spatial memory impairment, in transgenic mice. Recently [68], *ω*3 was shown to reduce cytokine-induced expression of proatherogenic and proinflammatory proteins, in human endothelial cells, and these properties may contribute to the antiatherogenic and anti-inflammatory effects of n-3 PUFAs.

In the present study, the treatment of ischemic animals with *ω*3 drastically decreased immunoreactivity for TNF-alpha in the hippocampus and temporal cortex. A previous work [69] demonstrated that *ω*3 pretreatments significantly attenuated LPS-stimulated TNF-alpha production. Others [70] showed that the inhibition by *ω*3 of proinflammatory cytokines as TNF-alpha, in murine macrophages, is in part mediated by the inactivation of NF-kappaB signaling. Besides, Nielsen et al., 2005 [71], observed that *ω*3 fatty acids were able to inhibit the increase in proinflammatory cytokines, in patients with Crohn's disease. *ω*3 fatty acids are known to affect immune response, partly by influencing cytokine secretion [72].

Furthermore, we also showed that the treatments with *ω*3 of ischemic animals significantly decreased COX-2 and iNOS immunoreactivity in hippocampal brain areas. Evidences indicate that *ω*3 fatty acids dampen inflammation, through multiple pathways, and directly or indirectly suppress the activity of nuclear transcription factors, as NF-kappaB, and reduce the production of proinflammatory enzymes and cytokines, including COX-2, TNF-alpha, and IL-1B [73]. NF-kappaB is one of the most important transcription factors involved in inflammatory response and upregulation of gene encoding of inflammatory cytokines, adhesion molecules, and COX-2. Evidences indicated that *ω*3 decreases expression of adhesion molecules and production of inflammatory cytokines and COX-2 metabolites, and a common mechanism would be the impact on the NFkB system [74]. This could well be the way *ω*3 is acting in our study.

Besides decreasing COX-2 immunostaining, *ω*3 attenuates hippocampal iNOS immunoreactivity. It has been shown that a diet rich in *ω*3 fatty acids decreases eNOS and iNOS in the diabetic kidney [75]. Interestingly, the attenuation of iNOS in LPS-stimulated macrophages by *ω*3 was shown to be independent of COX-2 derived PGE2, contradicting the hypothesis that the decrease in NO production associated with *ω*3 treatments occurs through a COX-2 derived PGE2 dependent mechanism [76]. The expression of iNOS is one direct consequence of inflammatory processes [77]. One important step during inflammation is leukocyte infiltration, mainly controlled by chemokines. The production of these chemokines is positively or negatively regulated by iNOS-derived NO [78]. Furthermore, a clinical study demonstrated that fish oil decreased serum levels of TNF-alpha, IL-1beta, IL-6, and nitric oxide metabolites in multiple sclerosis patients treated with IFN-1beta [79].

## 5. Conclusion

*ω*3 fatty acids by their anti-inflammatory and antioxidant properties that contribute to the neuroprotective property might be a therapeutic target to be pursued for the prevention or treatment of inflammation-related diseases. *ω*3 deficiency increases the brain's vulnerability, representing an important risk factor for the development of neuropathologies [80]. Furthermore, understanding the precise roles of *ω*3 fatty acids, as a possible disease modifier, will permit the development of new therapies for neurological diseases.
